# Relationships between fatigue, cognitive function, and upright activity in a randomized trial of oxaloacetate for myalgic encephalomyelitis/chronic fatigue syndrome

**DOI:** 10.3389/fneur.2025.1691147

**Published:** 2025-10-08

**Authors:** Suzanne D. Vernon, Candace Rond, Yifei Sun, Shad Roundy, Jennifer Bell, Bella Rond, David L. Kaufman, Alan B. Cash, Brayden Yellman, Lucinda Bateman

**Affiliations:** ^1^Bateman Horne Center, Salt Lake City, UT, United States; ^2^Department of Mechanical Engineering, University of Utah, Salt Lake City, UT, United States; ^3^Center for Complex Diseases, Seattle, WA, United States; ^4^Terra Biological LLC, San Diego, CA, United States

**Keywords:** myalgic encephalomyelitis, cognitive impaiment, up time, oxaloacetate, fatigue-cognition coupling, ME/CFS, long COVID, brain fog

## Abstract

**Background:**

Myalgic Encephalomyelitis/Chronic Fatigue Syndrome (ME/CFS) is a debilitating condition characterized by fatigue, cognitive impairment, and reduced physical function. Oxaloacetate (OAA), a metabolic compound with potential mitochondrial and neuroprotective effects, has shown promise in reducing fatigue symptoms in ME/CFS. However, the interrelationships between fatigue, cognitive performance, and physical activity and their responsiveness to treatment remain poorly understood in ME/CFS.

**Methods:**

This 90-day randomized, double-blind, controlled trial evaluated the effects of 2,000 mg/day OAA or a control of 2,000 mg rice flour in 82 adults with ME/CFS. Self-reported fatigue (Chalder Fatigue Questionnaire), cognitive function (DANA Brain Vital), and upright activity time (UP Time) were assessed at baseline and three follow-up visits. Linear mixed-effects models examined associations between fatigue severity and cognitive/physical function, with treatment group interactions. Responder status at the last visit (Visit 4) was classified based on ≥15% fatigue reduction and/or ≥10% cognitive improvement.

**Results:**

The OAA group showed greater cognitive improvement over time, with a significant between-group difference at Visit 3, 60 days into the trial, (*p* = 0.034) and trends at other visits. Higher fatigue was significantly associated with reduced cognitive gains in the OAA group (*β* = −0.34, *p* < 0.0001), but not in controls. UP Time increased modestly in the OAA group, reaching significance at Visit 2, day 30 (*p* = 0.044), though fatigue was not a strong predictor of UP Time in either group. At Visit 4, day 90, Global and Fatigue Only Responders were more frequent in the OAA group, while Cognitive Only Responders were more frequent in controls, though group differences did not reach statistical significance (*p* = 0.10).

**Conclusion:**

OAA supplementation was associated with improved cognitive performance and small improvement in UP Time in ME/CFS participants receiving OAA. Fatigue–cognition coupling was particularly strong in OAA-treated participants, suggesting a potentially targetable phenotype. These findings underscore the importance of multidimensional outcome measures in ME/CFS clinical trials and support the need for more research and trials of metabolic interventions in ME/CFS.

## Introduction

Myalgic Encephalomyelitis/Chronic Fatigue Syndrome (ME/CFS) is a debilitating, multisystem illness characterized by profound fatigue, post-exertional malaise (PEM), cognitive impairment, orthostatic intolerance, and a constellation of immune, autonomic, and metabolic abnormalities (1). Prior to the COVID-19 pandemic, an estimated 2.5 million individuals were affected in the United States alone, imposing a substantial personal and societal burden, with economic costs ranging from $17 to $24 billion annually (2–5). Since the pandemic, it is estimated ME/CFS prevalence has increased to 4.5% or approximately 15 million people in the US (6). Despite its impact, less than 10% of people get diagnosed and there are no approved treatments (7).

Emerging evidence suggests that ME/CFS is associated with abnormalities in cellular energy metabolism, particularly involving mitochondrial function and the tricarboxylic acid (TCA) cycle. Several studies have reported altered metabolomic results related to mitochondrial energetics and disturbances in lipid, fatty acid, and amino acid metabolism in ME/CFS patients (8–11). These findings support a growing recognition that metabolic dysfunction may underlie key pathophysiological features of the disease, including impaired energy production that impacts both physical function and cognition.

Oxaloacetate (OAA) is a naturally occurring intermediate of the TCA cycle and a critical metabolite involved in gluconeogenesis, amino acid synthesis, and the urea cycle. Supplementation with OAA has been shown in preclinical models to improve mitochondrial biogenesis, enhance insulin signaling, reduce neuroinflammation, and increase resistance to muscle fatigue (12, 13). An open-label pilot study in ME/CFS patients suggested that OAA was safe and reduced fatigue symptoms, with over 80% of participants reporting improvements in fatigue over 6 weeks of supplementation (14). A follow-on randomized controlled trial of OAA in ME/CFS found that the treatment group had more than a 25% reduction in fatigue compared to the control group with almost half of the treatment group experiencing a 63% average fatigue reduction (15).

Fatigue is a heterogeneous construct encompassing physical, mental, cognitive, emotional, central, peripheral, and post exertional fatigue exacerbation (16). However, few studies have examined how distinct fatigue domains differentially impact objective measures of physical and cognitive function. Furthermore, the relationship between physical and cognitive performance remains poorly understood in ME/CFS and may vary by treatment or disease state.

The present study evaluates associations between self-reported fatigue, UP Time, and cognitive reaction time in participants followed over the course of a 90-day randomized controlled trial of oxaloacetate. Using linear mixed-effects modeling, we assessed whether self-reported physical and cognitive fatigue predict changes in UP Time and cognitive reaction time, and whether these relationships differ by treatment group.

## Methods

### Study design

RESTORE ME was a randomized, controlled, double blinded clinical trial of an oral dose of 2,000 mg OAA or a control of 2,000 mg rice flour for 90 days. The trial was conducted at the Bateman Horne Center (BHC) in accordance with Good Clinical Practice and the Declaration of Helsinki and approved by the Institute of Regenerative and Cellular Medicine Institutional Review Board (IRCM-2022-318). This trial was registered at ClinicalTrials.gov (NCT05273372). All participants provided written informed consent at enrollment.

Over the 90-day trial, there were four in-person visits to BHC, Day 1, 30, 60 and 90. At each in-person visit, patients completed questionnaires and a cognitive assessment using the Defense Automated Neurobehavioral Assessment (DANA) Brain Vital app. At the end of each in-person visit, patients were given a fully charged wearable device that was worn on the ankle to continuously measure time upright and with feet on the floor for 7 days. Participants were provided with a mailer to send the wearable device back to BHC after it had been worn for 7 days. REDCap was the electronic data capture system for this trial (17).

### Participants

The RESTORE ME clinical trial has been previously described (15). Briefly, we enrolled 82 participants between 18 to 65 years of age diagnosed with ME/CFS, with stable state of illness in the preceding 3 months, and self-reported upright activity between 2 and 6 h per day. Study participants had to have a negative COVID-19 test at the baseline visit. Participants were excluded if there was an alternate medical or psychiatric illness that could explain the ME/CFS symptoms, or they had severe ME/CFS with less than 2 h of upright activity a day, active or uncontrolled co-morbidities or current treatment stimulants. Pregnant women, women who had given birth within the past 6 months, or women who were breast feeding were ineligible. Participation in another clinical treatment trial, or symptoms improving because of treatment intervention in the past 3 months was exclusionary.

### Intervention

OAA was given as 500 mg capsules. The control capsules contained 500 mg of rice flour. Participants were instructed to take two 500 mg capsules with breakfast and two 500 mg capsules with lunch each day for the duration of the trial. Participants were provided with a 30-day supply of OAA or control capsules at each in-person visit.

### Outcomes

Outcomes analyzed for this study included the Chalder Fatigue Questionnaire (CFQ) to assess self-report physical and cognitive fatigue domains as well as total fatigue scores (18), upright position with feet on the floor time (UP Time), and cognitive reaction time (DANA Brain Vital).

### Cognitive assessment

Upon arrival at the BHC, participants downloaded the DANA Brain Vital app to their smartphones (19). DANA Brain Vital is a test that includes three reaction time and information processing measurements: simple reaction time (SRT), procedural reaction time (PRT), and sustained attention or Go-No-Go (GNG) (20). Cognitive performance across the three subtests (SRT, PRT, and GNG) was combined into a single composite variable, cognitive efficiency, calculated as (accuracy × speed) × 60,000, where speed is the reciprocal of median reaction time. SRT is a simple reaction time task in which the user taps an orange target symbol as soon as it appears on the screen. PRT incorporates choice by having the user differentiate between two sets of characters: a 2, 3, 4, or 5 appear on the screen, and the user taps one of two buttons [2 or 3] or [4 or 5]. GNG is a forced choice measure of reaction time where either a gray foe or green friend appears on the screen. The user is instructed to tap the screen only when the gray foe appears. Participants completed DANA Brain Vital at each of the four in-person visits.

### UP Time

Participants were provided with a fully charged wearable device at each visit. The wearable measured the amount of time they were in an upright position (UP Time) where upright is defined as having lower legs vertical with feet on the floor (e.g., sitting in a chair with feet on the floor would be measured as upright). Participants were asked to wear the device on the outer side of their lower right ankle continuously for 7 days. The only time the wearable was removed was during a bath or shower when participants were instructed to place it in an orientation as if they were standing if showering, or as if they were lying down if bathing. At the end of 7 days, participants returned the wearable to the BHC via mail where the raw data was processed and stored.

The wearable used for this study was the MetaMotionS (MMS) device^1^ which consists of a 9-axis inertial measurement unit (IMU) (3-axis accelerometer, 3-axis gyroscope, 3-axis magnetometer), a barometer, temperature sensor, ambient light sensors, a microcontroller, onboard memory, and bluetooth wireless communication. UP Time is calculated entirely from the IMU and does not make use of other sensors. For this study, raw IMU data were sampled at 25 Hz and logged on the device. The data was downloaded via a wired connection after being returned to Bateman Horne Center. A detailed description of the hardware, data collection, and data management system for UpTime has recently been described (21). UP Time is calculated as the average amount of time spent upright over a 24-h period, starting at midnight. Thus, Day 1 includes a partial day as the participant is given the wearable sometime during the day. Day 1 is further abnormal because the participant must come into the clinic, which can result in an artificially high UP Time. Likewise, Day 7 may only contain a partial day as the participant takes off the device and mails it back. A prior study, following a similar protocol, verified that Day 1 and Day 7 have abnormally high (Day 1) and low (Day 7) UP Time readings (22). Therefore, an average UP Time following each visit was calculated as the average of Days 2 through 6.

### Statistical methods

To evaluate longitudinal relationships between self-reported fatigue, cognitive reaction time, and UP Time, we conducted a series of linear mixed-effects models (LMMs). This approach accounts for the repeated-measures structure of the data by modeling within-subject variation over time while adjusting for between-subject heterogeneity. Separate LMMs were fit for cognitive reaction time as measured by DANA Brain Vital or upright time as objective measured with UP Time. The primary predictors were self-report physical and cognitive CFQ subscales, and CFQ total fatigue. To examine whether associations between cognitive reaction time and UP Time varied by treatment, we also included a treatment group interaction term in a separate model. All models included a random intercept for each participant to account for individual baseline differences and control for intra-subject correlation across visits. Timepoint (visit) was not modeled as a fixed effect, as the objective was to assess within-subject relationships across repeated observations rather than change over time. All models were estimated using restricted maximum likelihood to provide unbiased estimates of variance components in mixed models. Each model included a fixed intercept and slope for the CFQ primary predictor, a subject-level random intercept, and for interaction models, a fixed effect for treatment group or responder category, and its interaction with the primary predictor. Model assumptions were evaluated using residual diagnostics to confirm linearity and homoscedasticity. Model outputs included fixed-effect coefficients with standard errors, 95% confidence intervals (CI), and *p*-values. A threshold of *p* < 0.05 was used to determine statistical significance. Only participants with complete data for the relevant outcome and predictor variables were included in each model. No imputation was performed, and missingness was assumed to be at random.

In addition, percent change from baseline (Visit 1) was calculated for DANA cognitive efficiency scores and UP Time at each follow-up visit. Between-group comparisons of percent change at each visit were conducted using independent two-sample t-tests. These analyses were performed to provide visit-level descriptive context complementary to the LLMs and to illustrate patterns of change at specific timepoints.

To evaluate clinically meaningful improvement, participants were classified into four responder categories at Visit 4 based on predefined thresholds for fatigue and cognitive performance. Fatigue response was defined as a ≥ 15% reduction from baseline in Chalder Fatigue Questionnaire (CFQ) total score, while cognitive response was defined as a ≥ 10% improvement from baseline in DANA Brain Vital composite score. These thresholds were selected based on prior conventions where a 10–15% change has been used as a minimal clinically important difference for self-reported and cognitive outcomes (19, 23, 24). Given the absence of standardized responder definitions in ME/CFS, we considered these criteria exploratory and complementary to the primary continuous outcome analyses. Participants who met both criteria were classified as Global Responders; those who met only the fatigue criterion were Fatigue Only Responders; those who met only the cognitive criterion were Cognitive Only Responders; and those who met neither criterion were classified as Minimal Responders. Responder frequencies were compared between the Oxaloacetate and Control groups using a chi-square test of independence, and standardized residuals were examined to assess the contribution of each category to group differences. Proportions were visualized to illustrate response profiles by treatment group. All statistical analyses were performed using Python (statsmodels v0.13.5) and were reviewed and verified by the corresponding author.

## Results

**Figure 1:**
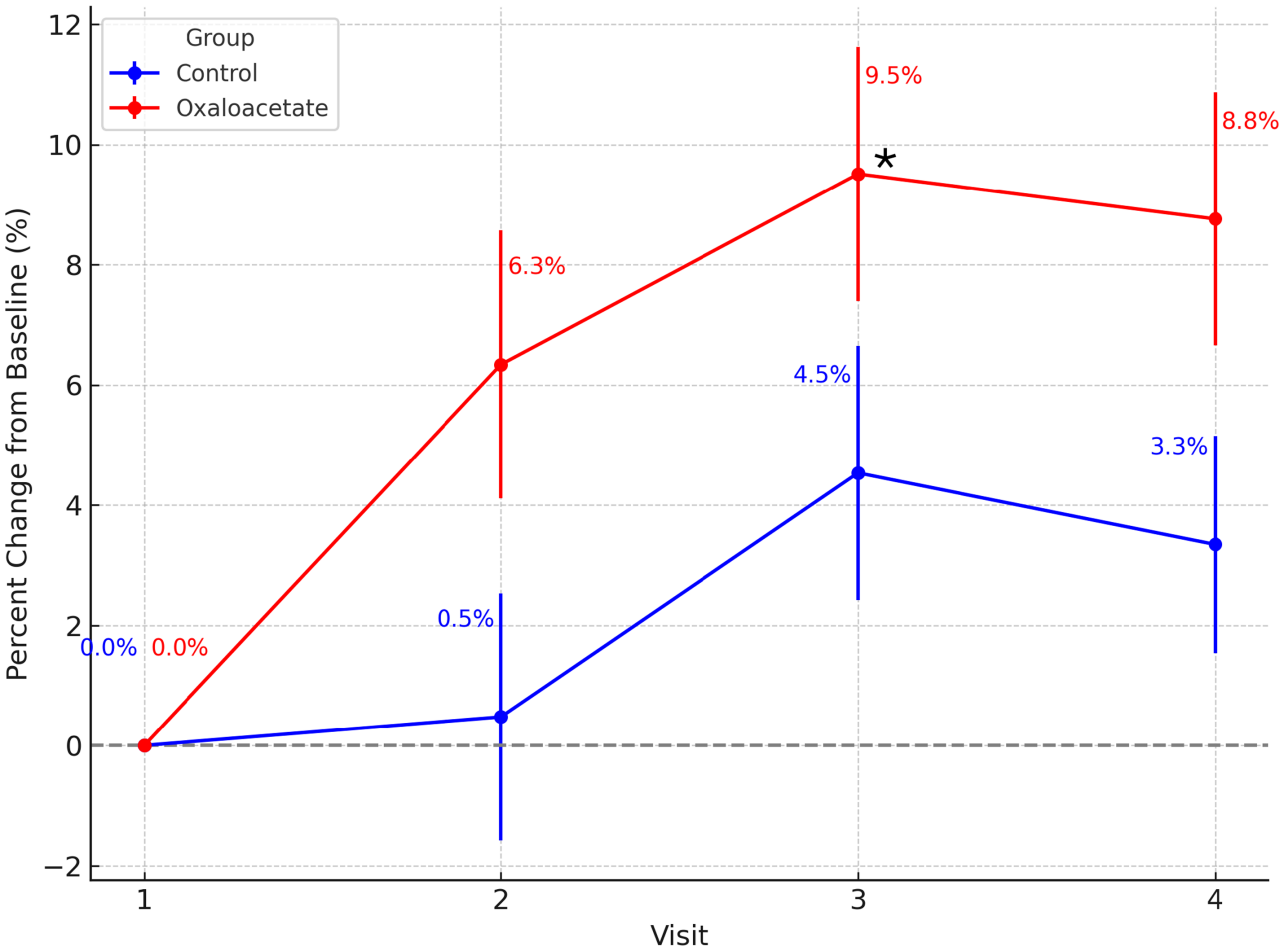
Percent change in DANA cognitive efficiency scores over time by treatment group. Percent change from baseline (Visit 1) is shown for the OAA (red) and control (blue) groups across Visits 2–4. Error bars represent standard errors of the mean. Group means are labeled at each visit, and asterisks indicate statistically significant between-group differences (*p* < 0.05). The OAA group demonstrated greater cognitive improvement relative to baseline, with a statistically significant difference compared to the control group at Visit 3 (*p* = 0.034), and trends toward significance at Visits 2 (*p* = 0.063) and 4 (*p* = 0.053).

Percent change analysis of DANA cognitive efficiency scores from baseline (Visit 1) revealed greater improvement over time in the OAA group compared to the control group (Figure 1). While both groups showed small cognitive gains, the Oxaloacetate group demonstrated consistently higher percent increases at each subsequent visit. The between-group difference reached statistical significance at Visit 3 (*p* = 0.034), with trends toward significance at Visits 2 (*p* = 0.063) and 4 (*p* = 0.053).

**Figure 2:**
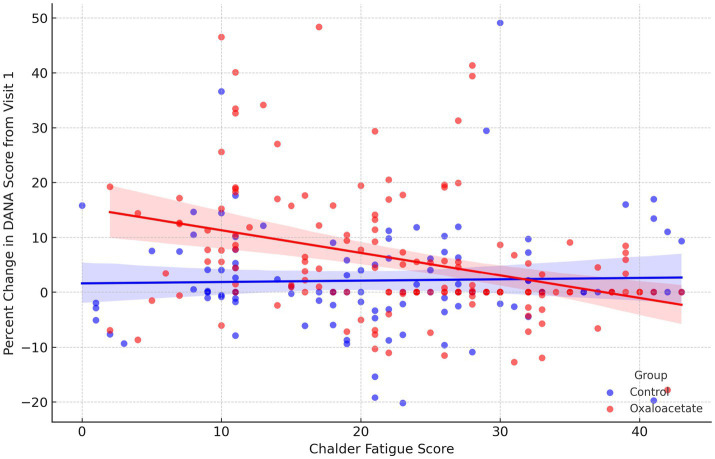
Relationship between fatigue severity and percent change in DANA cognitive scores, stratified by treatment group. Scatterplot showing the association between CFQ total scores and percent change in DANA cognitive efficiency scores from Visit 1, with regression lines shown for the OAA (red) and control group (blue). Each point represents an individual participant at a given visit. Shaded areas represent 95% confidence intervals. A significant inverse relationship was observed in the OAA group (*β* = −0.34, *p* < 0.0001), indicating that greater fatigue was associated with less cognitive improvement. No significant association was observed in the control group (*r* = 0.03, *p* = 0.77).

A linear mixed model was conducted to evaluate whether fatigue severity, as measured by the CFQ, predicted change in cognitive performance, and whether this relationship differed by treatment group. Percent change in DANA cognitive efficiency scores from baseline was modeled as the dependent variable, with CFQ total fatigue score, treatment group, and their interaction included as fixed effects, and participant as a random effect. The model revealed a significant group-by-fatigue interaction (*p* = 0.006), indicating that the association between fatigue and cognitive change varied by treatment group (Figure 2). In the OAA group, higher fatigue was significantly associated with reduced cognitive improvement (*β* = −0.34, *p* < 0.0001), whereas in the control group, no significant relationship was observed (*r* = 0.03, *p* = 0.77).

**Figure 3:**
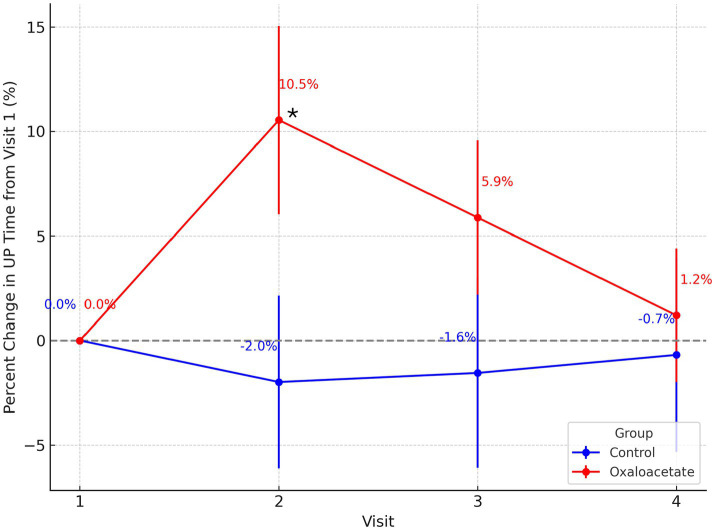
Percent change in UP Time from baseline across visits by treatment group. Percent change in UP Time from Visit 1 is shown for the OAA group (red) and control group (blue) across Visits 2–4. Group means are labeled at each timepoint, with error bars representing the standard error of the mean. The OAA group showed greater improvements in UP Time relative to baseline, reaching statistical significance at Visit 2 (*p* = 0.044), as denoted by the asterisk. While no other timepoints showed significant between-group differences (*p* > 0.10), the OAA group maintained higher percent gains compared to the control group throughout the follow-up period.

Percent change analysis of UP Time from baseline (Visit 1) revealed modest improvements in the OAA group compared to the control group across follow-up visits (Figure 3). The between-group difference in percent change reached statistical significance at Visit 2 (*p* = 0.044), with the OAA group showing greater increases in upright activity time. Although group differences at Visits 3 and 4 did not reach statistical significance (*p* > 0.10), the OAA group maintained numerically higher percent gains at each timepoint.

**Figure 4:**
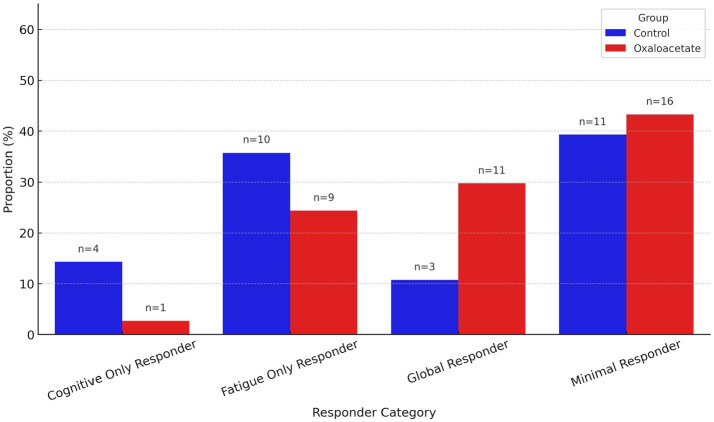
Proportion of participants in each responder category at Visit 4, stratified by treatment group. Responders were classified based on percent change from baseline: a ≥ 15% reduction in CFQ score indicated a fatigue response, and a ≥ 10% improvement in DANA Brain Vital score indicated a cognitive response. Categories include: Global Responders (meeting both criteria), Fatigue Only Responders, Cognitive Only Responders, and Minimal Responders (meeting neither criterion). Bars show the percentage of participants in each group; annotations indicate the number of participants (n) in each category. The OAA group is shown in red and the control group in blue.

A linear mixed model was used to evaluate whether fatigue severity, measured by the CFQ, predicted changes in UP Time across study visits. In the overall model, there was a trend-level association between higher fatigue and reduced UP Time (*β* = −0.062, *p* = 0.059). However, when stratified by treatment group, no statistically significant relationship was observed in either group. In the control group, the fatigue–UP Time association was small and non-significant (*β* = −0.059, *p* = 0.31), and in the OAA group, a similar non-significant trend was observed (*β* = −0.064, *p* = 0.095). These results suggest that fatigue severity was not a strong predictor of upright activity time within either group (Figure 4).

At Visit 4, participants were classified into four responder categories using predefined thresholds: a ≥ 15% reduction in CFQ scores and a ≥ 10% improvement in DANA Brain Vital scores. In the OAA group (*n* = 22), 45.5% were Minimal Responders, 31.8% were Fatigue Only Responders, 22.7% were Global Responders, and only 1 (5%) was a Cognitive Only Responder. In the control group (*n* = 22), 36.4% were Minimal Responders, 27.3% were Cognitive Only Responders, 22.7% were Global Responders, and 13.6% were Fatigue Only Responders. A chi-square test comparing responder distributions between groups was not statistically significant (*χ*^2^ = 6.22, df = 3, *p* = 0.10).

## Discussion

In this randomized controlled trial of OAA for ME/CFS, we observed significant and clinically relevant improvements in cognitive function and small increases in upright activity among participants receiving OAA over a 90-day period. The data further revealed a strong inverse relationship between self-reported fatigue and cognitive performance in the OAA group, suggesting that the coupling between fatigue severity and neurocognitive function may represent a modifiable and treatment-responsive phenotype in ME/CFS.

Notably, cognitive performance, as measured by DANA Brain Vital, improved more in the OAA group compared to the control group, with statistically significant gains emerging at Visit 3 (day 60) and consistent trends at other visits. Importantly, this cognitive enhancement was not uniform but instead appeared tightly linked to individual fatigue levels in the OAA group. Participants with higher fatigue scores showed smaller improvements in cognitive function, a relationship that was not present in the control group. This differential pattern suggests that OAA may modulate fatigue-related cognitive impairments through mechanisms not engaged by the control. At the same time, the strong inverse association between fatigue and cognition raises the possibility that cognitive benefits are, at least in part, downstream effects of reduced fatigue. Preclinical studies, however, also support direct neuroprotective and metabolic effects of OAA on brain function, including improved mitochondrial energetics, reduced neuroinflammation, and enhanced oxidative stress resistance. While our study design does not allow us to disentangle direct versus indirect pathways, the data indicate that both mechanisms may plausibly contribute to the observed improvements.

Time upright with feet on the floor, measured by average daily upright time (UP Time), also increased in the OAA group, with a statistically significant group difference observed at Visit 2 (day 30). While these gains were more modest and did not persist to significance at later visits, the consistently higher UP Time in the OAA group across all follow-ups suggests a possible sustained benefit in real-world physical function. However, fatigue was only marginally predictive of UP Time, and no clear interaction by treatment group emerged, indicating that UP Time may be influenced by additional factors such as orthostatic intolerance, post-exertional malaise, or pacing, hallmarks of ME/CFS that are not easily captured by self-report fatigue scales.

The responder analysis provided further insights into individual-level patterns of benefit. Participants receiving OAA were more likely to be classified as Fatigue Only or Global Responders, whereas Cognitive Only Responders were more prevalent in the control group. Although these group differences did not reach statistical significance, they align with the primary analyses showing stronger fatigue–cognition coupling in the OAA arm. This subgroup variability highlights the value of integrated outcome modeling to detect meaningful within-subject changes, particularly in complex, heterogeneous conditions like ME/CFS. It should be noted that the responder definitions were exploratory, and dichotomizing continuous outcomes may reduce statistical power and obscure more subtle effects. Nonetheless, these thresholds provided a pragmatic framework to explore individual-level patterns of treatment response in parallel with the continuous models. Identifying treatment-responsive phenotypes, such as those exhibiting concurrent improvement in both fatigue and cognition, may be essential for advancing precision therapeutics.

Several strengths bolster the credibility of our findings. The use of continuous, objective monitoring of upright activity time and a validated, mobile cognitive assessment tool allowed for high-resolution measurement of key functional domains. Linear mixed modeling accounted for within-subject variability and provided robust estimates of dynamic relationships over time. However, several limitations warrant caution. The relatively short trial duration may have limited the detection of delayed treatment effects, particularly for physical function. The modest sample size, while adequate for modeling repeated measures, may have underpowered some between-group comparisons, such as the responder classification.

In conclusion, our results support the therapeutic potential of oxaloacetate in ME/CFS, particularly in improving cognitive efficiency in a subset of individuals with high fatigue. The observed fatigue–cognition interaction may serve as a biomarker for identifying individuals most likely to benefit from metabolic interventions. Future trials should aim to replicate these findings in larger cohorts and explore mechanistic correlates of treatment response, with a focus on integrating multidimensional outcomes to capture the complexity of ME/CFS.

## Data Availability

The raw data supporting the conclusions of this article will be made available by the authors, without undue reservation.

## Footnotes

^1^Mbient Lab Inc., https://mbientlab.com/metamotions/
